# Effect of Changes in Dietary Net Energy Concentration on Growth Performance, Fat Deposition, Skatole Production, and Intestinal Morphology in Immunocastrated Male Pigs

**DOI:** 10.3389/fvets.2021.789776

**Published:** 2021-12-14

**Authors:** Nina Batorek-Lukač, Marjeta Čandek-Potokar, Martin Škrlep, Valentina Kubale, Etienne Labussière

**Affiliations:** ^1^Agricultural institute of Slovenia, Animal Production Department, Ljubljana, Slovenia; ^2^Veterinary Faculty, Institute of Preclinical Sciences, University of Ljubljana, Ljubljana, Slovenia; ^3^PEGASE, INRAE, Institut Agro, Saint Gilles, France

**Keywords:** immunocastration, precision feeding, dietary fiber, leanness, energy reduction, porc

## Abstract

Nutritional requirements of heavy immunocastrated (IM) pigs and therefore appropriate feeding strategies have not yet been determined. Thus, the effects of changes in dietary net energy (NE) content were studied in 41 IM pigs, fed *ad libitum* diets with low, medium, and high NE content (LNE, MNE, and HNE diets, with 8.5, 9.3, and 10.0 MJ NE/kg, respectively), from 84 days of age until slaughter at an average age of 172 days and an average body weight of 122.5 kg. In the period from 143 to 170 days of age, there was a tendency for a greater NE intake (*p* = 0.08) in pigs fed the HNE diet along with greater (*p* < 0.01) backfat gain. Dietary treatment affected carcass composition, as lower backfat thickness (*p* = 0.01) and lower area of fat over the longissimus muscle (*p* = 0.05) were observed in the LNE and MNE pigs. In addition, greater lean meat content (*p* = 0.04) was observed in the LNE pigs. Reducing the NE of the diet by replacement of cereals and soybean meal with high-fiber ingredients resulted in lower indole production in the ascending colon (*p* < 0.01) and greater skatole production (*p* < 0.01) in the cecum. Greater villus area, width, height and perimeter, crypt depth, and thickness of the intestinal mucosa in the jejunum, ileum, ascending colon, and descending colon were found in the LNE group (*p* < 0.01) than in the HNE group, while those in the MNE group was intermediate. Cell proliferation was not affected by dietary treatment (*p* > 0.05). The present results show that a reduction in dietary NE concentration lowers lipid deposition, without affecting performance or energy efficiency in IM pigs. This technique provides an advantage in terms of improved leanness, without affecting growth rate in IM pigs after immunization, which is particularly important when the backfat thickness is a determinant of carcass value and IM pigs are fattened to higher weights (e.g., in heavy pig production) or when a longer delay between immunization and slaughter is practiced.

## Introduction

In many countries, male piglets are still surgically castrated shortly after birth as a preventive measure against the development of boar taint. Lately, the vaccination against gonadotropin-releasing hormone (i.e., immunocastration) has been proposed as an alternative to surgical castration that improves animal welfare and allows to benefit from lean tissue growth and feed efficiency of entire males (EM) for most of the growing period (1). When standard immunization protocols are used, the physiological effects of immunocastration become evident as early as 1 week after effective immunization [i.e., after applying the second dose of vaccine (2)]. However, after effective immunization, a substantial increase in daily feed intake (DFI) is observed in immunocastrated (IM) pigs, resulting in reduced feed efficiency and increased body fatness as compared with EM (3, 4). These negative consequences can be limited either by restricting feed allowance after immunization (5) or by shortening the time interval between immunization and slaughter, which gives pigs less time to deposit fat (6). Quantitative feed restriction has been shown to improve the performance of IM pigs (5) but may also stimulate aggressive behavior and increase the level of stress (5, 7), raising questions about welfare degradation. As an alternative, a reduced energy concentration of the *ad libitum* diet may be considered. Since the energy component of the feed accounts for most of the cost in pork production (8), replacing energy-rich starch from cereals with relatively inexpensive fibrous feedstuffs, such as cereal by-products, in the finishing diet may be of interest for IM pigs. Additionally, dietary fiber may be beneficial for physiological function and gastrointestinal health (9). Immunocastration has been shown to effectively prevent the accumulation of boar taint compounds (androstenone and skatole) in adipose tissue by reducing steroid hormones synthesis in the testes and also by preventing the inhibition of skatole degradation by steroids in the liver (10). However, skatole production in the intestine is influenced by numerous other factors including housing conditions (11), animal health (12), and dietary composition (13, 14). Namely, by influencing the rate of apoptosis, diet can alter the availability of l-tryptophan, the precursor of skatole, in the colon and thus can affect skatole production, while skatole absorption can be reduced by accelerating the intestinal transit time (13, 14).

Limiting (excessive) fat deposition after immunocastration by dietary measures may become a key factor for the economic sustainability of this alternative especially when pigs are slaughtered at a greater age and weight (e.g., in special production systems). Hence, the objective of this study was to evaluate the effect of net energy (NE) concentration of the feed given to IM pigs during the prolonged period between effective vaccination and slaughter on growth performance, fat deposition, skatole production, intestinal morphology, and cell proliferation.

## Materials and Methods

### Animals and Experimental Design

**Figure 1:**
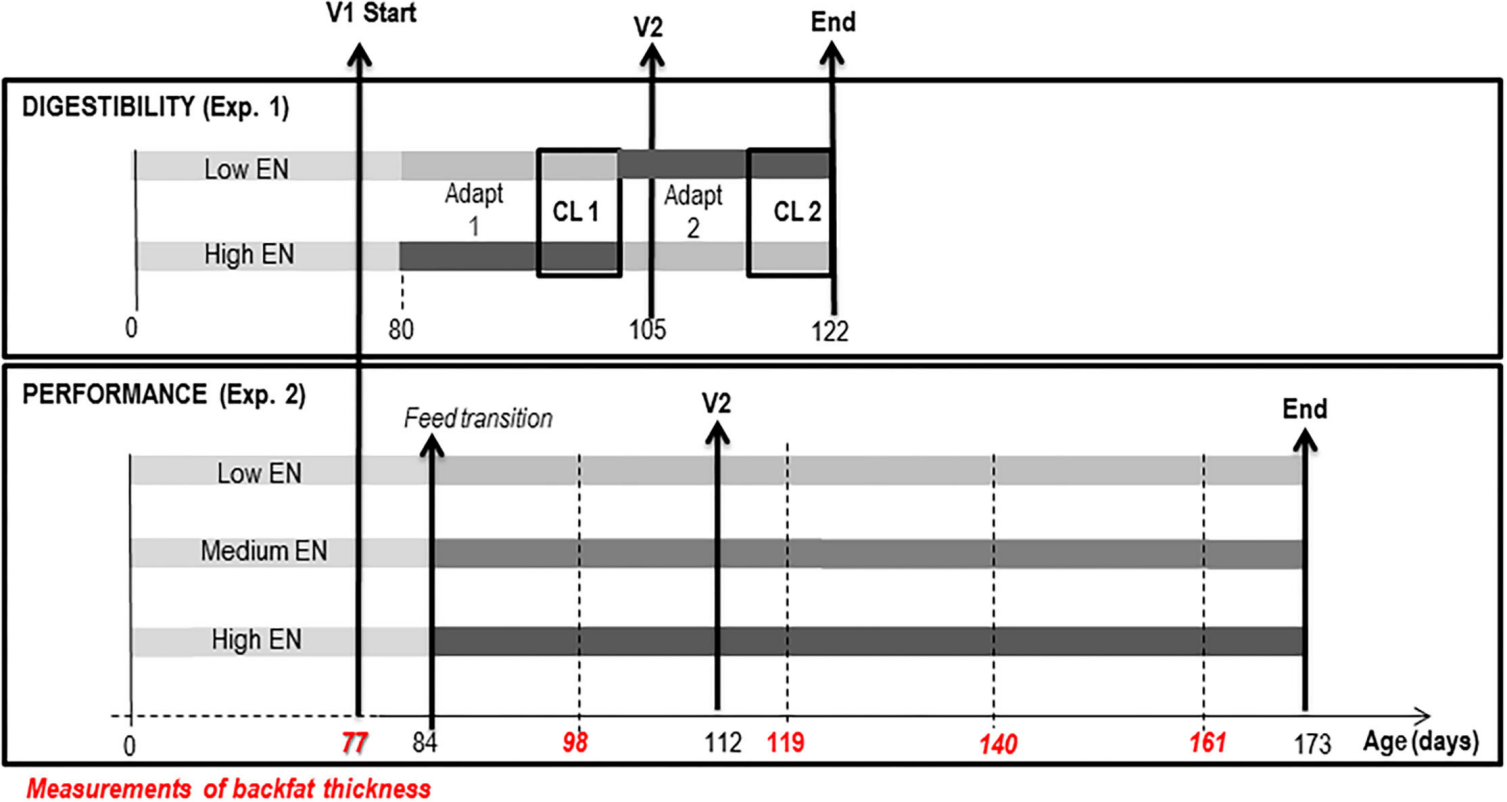
Experimental design of Experiments 1 and 2. Digestibility and nitrogen balance (Exp. 1) was performed in 2 consecutive periods, composed of adaptation (Adapt 1 and 2) and collection period (CL 1 and CL 2), on each of the 4 pigs fed the 2 extreme diets in order to calculate the net energy (NE) content of the 3 diets. Net energy content of the Medium NE diet was calculated as the average of the NE content of Low NE and High NE diets. The performance experiment (Exp. 2) included 45 immunocastrated pigs (immunocastrated at the age of 77 and 112 days and slaughtered at the age of 172 days), divided into 3 experimental groups, each receiving one of the diets with low (Low NE), medium (Medium NE), or high (High NE) net energy (NE) content.

As presented in Figure 1, the study included an experiment on informative digestibility and nitrogen balance (Exp. 1) and a growth experiment (Exp. 2). The experiments aimed to compare the utilization by IM pigs of three cereal-based diets differing in their NE content (Table 1). To this end, the NE content was reduced from 10.0 MJ/kg in the high NE diet (HNE) to 9.3 and 8.5 MJ/kg in the medium NE (MNE) and low NE (LNE) diets, respectively, by substitution of cereals (wheat, barley, and corn) and soybean meal with high-fiber ingredients (wheat bran, soybean hulls, and dried beet pulp) and reduction of the amount of added sunflower oil. The MNE diet was calculated as the mean composition of HNE and LNE diets.

**Table 1:** Ingredients and chemical composition of experimental diets fed to immunocastrated male pigs.

	**Diet** ^*****a*****^		
**Item**	**LNE**	**MNE**	**HNE**
**Ingredient composition (%)**			
Wheat	17.77	21.35	24.93
Corn	17.77	21.35	24.93
Barley	17.77	21.35	24.93
Wheat bran	15.00	8.75	2.50
Soybean hulls	10.00	5.00	-
Dried beet pulp	5.00	2.50	-
Soybean meal	9.18	12.46	15.74
Rapeseed meal	1.97	0.99	-
Cane molasses	3.00	3.00	3.00
Sunflower oil	-	0.50	1.00
l-Lysine HCl	0.24	0.27	0.29
l-Threonine	0.07	0.08	0.09
l-Tryptophan	0.01	0.01	0.02
dl-Methionine	0.01	0.02	0.03
l-Valine	-	0.01	0.02
Sodium chloride	0.45	0.45	0.45
Limestone	0.29	0.41	0.54
Dicalcium phosphate	0.97	1.00	1.03
Vitamins–minerals premix^*b*^	0.50	0.50	0.50
**Measured chemical composition, g/kg of DM**			
Ash	60	56	54
Crude protein (N × 6.25)	180	175	185
Starch	416	470	518
Ether extract	33	34	42
Crude fiber	80	60	34
Neutral detergent fiber	228	188	147
Acid detergent fiber	100	74	44
GE, MJ/kg of DM	18.17	18.17	18.31
**Nutritional values** ^***c***^			
DE, MJ/kg of feed	12.24	13.00	13.89
NE, MJ/kg of feed	8.50	9.25	9.99
SID lysine^*d*^, g/kg of feed	7.1	7.8	8.4
SID threonine, g/kg of feed	4.6	5.0	5.4
SID tryptophan, g/kg of feed	1.4	1.5	1.8
SID methionine, g/kg of feed	2.0	2.3	2.6
SID valine, g/kg of feed	5.4	5.9	6.5

*Experimental diets were fed to immunocastrated male pigs between 84 and 172 days of age. Immunocastration with Improvac® (2 ml, s.c. application, Zoetis) was performed at the age of 77 and 112 days. DM, dry matter; GE, gross energy; DE, digestible energy; NE, net energy; SID, standardized ileal digestible*.

a *Diets with low (LNE), medium (MNE), or high (HNE) NE content*.

b *Supplied per kilogram of final diet (as-fed basis): vitamin A, 5,000 UI; vitamin D3, 1,000 UI; vitamin E, 20 UI; vitamin B1, 2 mg; vitamin B2, 4 mg; pantothenic acid, 10 mg; vitamin B6, 1 mg; vitamin B12, 0.02 mg; niacin, 15 mg; vitamin K3, 2 mg; folic acid, 1 mg; biotin, 0.2 mg; choline chloride, 500 mg; iron, 80 mg; copper, 10 mg; zinc, 100 mg; magnesium, 40 mg; cobalt, 0.1 mg; iodine, 0.2 mg; selenium, 0.15 mg*.

c *Calculated from the feed ingredients according to Sauvant et al. (15), adjusted to 89% DM content of the diet*.

d *The SID lysine/NE ratio was constant among diets at a level of 0.84 g/MJ*.

### Experiment 1

To accurately calculate the NE content (8), a digestibility and nitrogen balance study was conducted with 4 IM pigs (Improvac® 2 ml, subcutaneous application at 77 and 105 days of age, Zoetis Florham Park, NJ, USA). Pigs [Piétrain × (Large White × Landrace)] were housed individually in digestibility cages and fed the two extreme experimental diets (i.e., HNE and LNE) for two consecutive periods, such that there were two pigs per diet in each period. Each period consisted of an adaptation sub-period (14 days, allowing pigs to adapt to the feed, digestibility cage, and environmental conditions) and a subsequent total collection sub-period (7 days), during which feed intake was recorded and feces and urine were separately collected. Pigs were weighed at the beginning (at 77 days of age) of the trial and at the beginning and the end of each collection sub-period. During the collection period, feed allowance was restricted to 90% of predicted *ad libitum* NE intake [calculated according to INRA Porc software (16)]. Water was freely available. Pelleted feed was prepared daily and distributed in equal amounts 3 times per day (09:00, 12:00, and 15:00), whereas feed residues were collected each morning and stored at 4°C until analysis of dry matter (DM; 24 h in a ventilated oven at 103°C). A representative sample of each diet was collected during the collection period and stored at 4°C until analysis of DM and chemical composition. During the collection period, feces and urine (collected in a 10-L plastic container with 120 ml of 10% sulfuric acid) were collected each morning. Feces were stored at 4°C and accumulated during the collection period. On the day following the last collection, total collected feces were weighed and homogenized. Three representative samples were taken, two of which were dried for 48 h at 100°C for determination of DM, while the third sample was freeze-dried, ground (1-mm grind), and stored at 4°C until further laboratory analyses. The containers for urine collection were changed every morning, and the pH was measured and, if necessary, adjusted by adding 10% sulfuric acid, so that the value was below 2.0. Afterward, urine was weighed and homogenized, and a representative sample (1%) was taken, cumulated over the entire collection period, and stored at 4°C.

### Experiment 2

At 70 days of age, 45 male piglets [Piétrain × (Large White × Landrace)] from 12 different litters were transferred to the experimental stables (individual housing, concrete floor, and water freely available) and fed a commercial growing diet until the start of the experiment. Two vaccinations with Improvac® (2 ml, subcutaneous application, Zoetis Florham Park, NJ, USA) were given at 77 and 112 days of age. At 84 days of age, the pigs (within the litter) were allocated to 3 groups of 15 animals receiving one of the experimental diets (i.e., HNE, MNE, and LNE) in pelleted form, with a progressive transition that lasted for 5 days. During the experiment, pigs were fed individually 3 times per day at 09:00, 12:00, and 15:00 (manual feeding in the individual trough). Daily meals were prepared in buckets containing the expected *ad libitum* feed intake for 3 or 4 days plus 10% [calculated according to INRA Porc software (16)]. At the end of each 3- or 4-day period, accumulated feed refusals were collected and weighed. Feed refusals were not contaminated by urine or saliva and were therefore not dried, and their DM content was considered as for the offered feed, which was measured at each feed preparation.

Pigs were weighed weekly at the same time in the morning, without prior limitation of feed allowance. Ultrasound measurements (Noveko, VetkoPlus, 3.5 MHz) of the backfat thickness (BFT) at the level of the last rib (measured on the left and right sides of the back, 6 cm lateral to the spine and subsequently averaged per pig) were performed every 3 weeks from the first vaccination (V1) onwards to obtain 5 consecutive measurements on each animal during the experiment. The difference in BFT divided by the number of days elapsed between the corresponding measurements was calculated to assess the daily backfat gain in the total and interim periods.

Pigs were slaughtered (at an average age of 172 days) in two batches, balanced according to experimental groups and litter of origin, within a week in the experimental slaughterhouse of INRAE Saint-Gilles following standard slaughter procedures (approximately 20 h of feed withdrawal, electrical stunning, and immediate vertical bleeding). At evisceration, samples of the duodenum (at the caudal end of the pancreas), jejunum (the middle part), ileum (10 cm anterior to the ileocecal valve), cecum (the second haustrum distal to the apex), ascending colon (the top of the spiral colon), and descending colon (30 cm from the end of the intestine) were collected and stored in 5% buffered formalin. At the same time, the intestinal contents from the cecum, ascending colon, and descending colon were collected, immediately frozen in liquid nitrogen, and stored at −20°C until analysis. At the end of the slaughter line, hot carcass weight (HCW) was recorded, and testicles were removed, dissected, and weighed. The weight of both testicles (including epididymis) was recorded for each pig. Leaf fat was excised from both half-carcasses and weighed.

Carcasses were chilled overnight until the internal carcass temperature was below 7°C. Additional carcass characteristics were assessed the following day. Intermuscular neck fat (percent), longissimus muscle area (square centimeters), and fat over longissimus muscle area (square centimeters) were assessed by image analysis on pictures of carcass cross sections taken between the 3rd and 4th cervical vertebrae (for evaluation of intermuscular neck fat) and after the last rib (for evaluation of longissimus muscle area and fat over longissimus muscle), as described by Batorek et al. (5).

Caudally from the level of the last rib, a 2.5-cm-thick slice of longissimus muscle was removed, vacuum-packed, and stored at −20°C to determine intramuscular fat using NIRS (NIR System model 6,500 Spectrometer, Silver Spring, MD, USA) and in-house developed calibrations (Agricultural Institute of Slovenia). For determination of boar taint compounds, samples of subcutaneous fat were taken at the level of the last rib, vacuum-packed, and stored at −20°C until further laboratory analyses.

### Chemical Analyses

Pooled feed samples from Exp. 1 and 2 were analyzed for DM, ash, nitrogen, starch, crude fiber, and ether extract contents according to the Association of Official Analytical Chemists (AOAC) (17). Gross energy (GE) content was measured by an adiabatic bomb calorimeter (IKA, C5000, Staufen, Germany). The content of neutral detergent fiber (NDF) and acid detergent fiber (ADF) of pooled samples of diets was analyzed according to Van Soest et al. (18). Samples of feces from Exp. 1 were analyzed for DM, ash, nitrogen, crude fiber, ether extract with prior acid hydrolysis, NDF, ADF, and GE contents using the same methods as for feed samples. Fresh samples of urine from Exp. 1 were analyzed for nitrogen content, while the GE content was evaluated after freeze-drying of approximately 30 ml of urine in polyethylene bags.

Androstenone and skatole concentrations were measured in subcutaneous fat samples from Exp. 2 by high-performance liquid chromatography (HPLC) (5) and expressed per gram of liquid fat. The detection limits were 0.24 and 0.03 μg/g for androstenone and skatole, respectively. Skatole and indole concentrations in intestinal content samples from Exp. 2 were measured by a modified method of Denhard et al. (19, 20). Concentrations were expressed per gram of sample, and the detection limit was 1.12 μg/g for skatole and indole.

### Histological and Immunohistochemical Evaluations of Intestinal Segments

Tissue samples from the intestinal segments collected in Exp. 2 were embedded in paraffin, and histological sections (5 μm thick) were cut and stained with H&E. Morphometric analysis was performed as described in detail by Bilič-Šobot et al. (21) using a Nikon Microphot FXA microscope equipped with a DS-Fi1 camera and the Imaging Software NIS Elements D.32 (Nikon Instruments Europe B.V., Badhoevedorp, The Netherlands). For each tissue sample of the small intestine, 20 villi and 20 crypts were examined at × 100 magnification for villus width, villus height, villus perimeter, and crypt depth. Additionally, villus surface area, thickness of the intestinal mucosa, and the ratio between villus height and crypt depth were calculated (21). For each tissue sample of the cecum and colon, 20 crypts were examined at × 100 magnification for crypt depth. To assess the extent of cell proliferation of the intestinal epithelium, immunohistochemical staining with mouse anti-proliferating cell nuclear antigen (anti-PCNA) as described in detail by Bilič-Šobot et al. (21) on all samples using the Dako REAL™ EnVision detection system (Dako, Glostrup, Denmark) was performed. In the small intestine segments, the number of PCNA-positive enterocytes was counted at the level of the villus–crypt boundary, which encompasses the upper crypt/lower villus region at a length of 150 μm (10 villus–crypt units per animal) at × 40 objective magnification. In the colon segments, the number of PCNA-positive enterocytes was counted in 10 well-oriented crypts per animal at × 20 objective magnification.

### Calculations

DM feed intake and apparent digestibility coefficients of organic matter (OM), crude protein (CP), GE, ether extract, crude fiber, NDF, and ADF were calculated from the values obtained in Exp. 1 using the total collection method. The NE content of the diet was calculated for HNE and LNE according to Noblet et al. (8) (using the average of the values obtained by equations 3, 4, and 5, with digestible energy (DE) as predictor), and the NE content of the MNE diet was assumed to be the average of values calculated for HNE and LNE diets. In Exp. 2, average daily gain (ADG) and gain to feed ratio (G:F) were calculated for each of 4-week experimental periods: day 84 to 114, day 115 to 142, and day 143 to 170. Additionally, DE, metabolizable energy (ME), and NE intake were calculated from DM feed intake and corresponding energy concentration of experimental diets as estimated in Exp. 1 and used in the calculation of body weight (BW) gain to NE intake ratio (G:NE). The energy values of experimental diets, average DFI (ADFI), and G:F were calculated on 100% DM and subsequently adjusted to 89% DM content. Dressing percentage was calculated as the ratio between HCW and final BW, measured 24 h before slaughter without prior fasting. Gonadosomatic index (%) was calculated as testicular weight (weight of right and left testes including epididymis) divided by final BW.

### Statistical Analysis

ANOVA was performed using the mixed model procedure of SAS (PROC MIXED, SAS Inst., Cary NC, USA). Repeated measurements of digestibility and nitrogen balance data (*n* = 8) in Exp. 1 were evaluated by including the fixed effects of diet group and stage and their interaction and the random effect of pig (included in the repeated statement). In Exp. 2, the effect of the diet (NE concentration) on growth performance was evaluated considering the fixed effects of dietary treatment and litter for the phases before (day 84 to 114) and after immunization (day 115 to 170). For the phase after the effective immunization, two 4-week periods were looked at separately, and the model included dietary group, period, their interaction, and litter, with a pig as a repeated variable. Because the effect of immunocastration on growth performance is now well established and was not the focus of the present study, comparisons of phases before and after immunization periods are not presented.

To predict the voluntary energy intake, data from the DM feed intake measurements per week, energy (ME and NE) content of the diets, and BW from the age of 84 days onwards were used to calculate relationships between cumulative energy intakes (y; ME or NE) and BW according to the following model:

If age < 112 days:

$y=a_{diet\;before\;V2}\times BW^{c_{diet}}+b_{pig}\;$

If age ≥ 112 days:

$y=a_{diet\;after\;V2}\times BW^{c_{diet}}+\left(a_{diet\;before\;V2}-a_{diet\;after\;V2}\right)\times BW_{age\;=\;112}+b_{pig}\;$

a_diet_ _beforeV2_ and a_dietafterV2_ are the slopes of the relationship for each diet, before and after immunization (V2), respectively; c_diet_ is the exponent applied to BW for each diet; BW_age_ = 112 is the BW at 112 days of age; and b_pig_ is the intercept of the relationship for each pig (so that cumulative intake of experimental diets equals zero at the start of the experiment, i.e., at 84 days of age). The parameters of the model were evaluated on the cumulative intakes using the NLIN procedure of SAS (PROC NLIN, SAS Inst., Cary NC, USA) to avoid threshold effects caused by the way feed intake was measured (continuous access to the feeder without restriction during the few hours preceding measurement). The hypothesis for a single c_diet_ exponent applied to BW for all diets was tested for ME and NE intake using the extra sum of squares reduction test (22). As this test was never significant (*p* > 0.10), a single exponent (1.43) was considered for further statistical analyses to test the effect of diet and period (P1 = before and P2 = after V2) on the regression coefficients (slopes) of the relationship.

Carcass trait, boar taint compounds, and testicular weight in Exp. 2 were analyzed using a fixed-effects model of diet, treatment group, litter within slaughter batch, and slaughter batch.

Measurements of morphology and mitosis cell count of small and large intestinal segments in Exp. 2 were evaluated by including the fixed effects of a dietary group within the intestinal part and the random effect of the pig.

The individual pig was considered as the experimental unit for all data analyses. The least-squares means were compared using Tukey's test with statistical significance based on *p* < 0.05.

## Results

The chemically determined values for the dietary CP, crude fiber, ether extract, and starch concentrations in the representative samples of the LNE, MNE, and HNE diets (Table 1) were as expected. No particular problems occurred in Exp. 1. In Exp. 2, one pig died during the experiment (the HNE group), two were excluded due to umbilical hernia (1 in the LNE group and 1 in the MNE group), and one was excluded due to broken forelimb (the MNE group), providing 41 pigs weighing on average 122.5 kg at the end of Exp. 2 (Table 2).

**Table 2:** Effect of dietary net energy concentration on feed intake and growth performance in immunocastrated pigs.

		**Treatment group**				***p*** **-Value** ^**1**^	
**Item**		**LNE**	**MNE**	**HNE**	**RSD^**2**^**	**Diet**	**Diet × Phase**
*n* of pigs		14	13	14			
**BW, kg**							
Day 84		33.6	32.8	32.8	3.5	0.80	-
Day 114		55.1	54.5	55.9	7.0	0.88	-
Day 142		85.8	86.0	89.4	10.4	0.61	-
Day 170		120.8	120.0	126.8	11.0	0.24	-
**NE intake** ^**3**^ **, MJ/day**							
Before V2:	Day 84 to 114	19.2	18.6	19.8	2.5	0.47	-
After V2:	Day 115 to 142	30.7	30.6	32.1	3.2	0.08	0.09
	Day 143 to 170	42.4	41.0	46.3			
	Day 115 to 170	36.6	35.6	39.2	8.0	0.24	-
**NE intake**^**4**^**, MJ/kg BW**^**0.60**^ **per day**							
Before V2:	Day 84 to 114	2.16	2.13	2.25	0.12	0.08	-
After V2:	Day 115 to 142	2.49^A^	2.46^A^	2.58^A^	0.20	0.04	0.40
	Day 143 to 170	2.67^AB^	2.58^AB^	2.84^B^			
	Day 115 to 170	2.58^ab^	2.52^a^	2.71^b^	0.25	0.03	-
**Average daily gain** ^**5**^ **, kg/day**							
Before V2:	Day 84 to 114	0.75	0.76	0.76	0.11	0.94	-
After V2:	Day 115 to 142	1.09	1.11	1.15	0.14	0.17	0.61
	Day 143 to 170	1.27	1.21	1.31			
	Day 115 to 170	1.18	1.16	1.23	0.16	0.27	-
**Gain to NE intake ratio** ^**4**^ **, g BW/MJ NE**							
Before V2:	Day 84 to 114	38.6	40.6	38.9	3.1	0.21	-
After V2:	Day 115 to 142	35.2	36.7	36.5	10.6	0.48	0.35
	Day 143 to 170	29.8	30.1	29.0			
	Day 115 to 170	32.5	33.4	32.7	4.4	0.76	-
Initial backfat thickness (day 84), mm	5.0	4.9	5.0	0.4	0.64	-
**Backfat gain, mm/day**							
Before V2:	Day 84 to 114	0.07	0.07	0.07	0.02	0.79	-
After V2:	Day 115 to 142	0.10^A^	0.11^A^	0.12^A^	0.04	<0.01	0.01
	Day 143 to 170	0.21^B^	0.22^B^	0.29^C^			
	Day 115 to 170	0.16	0.17	0.20	0.09	0.13	-

*Effect of dietary net energy concentration on feed intake and growth performance in growing–finishing immunocastrated pigs was evaluated during three successive 4-week phases of growth (Exp. 2). Vaccination with Improvac® (2 ml, s.c. application, Zoetis) was performed at the age of 77 and 112 days. Growth performance data were obtained over 3 successive 4-week phases of growth (84 to 114, 115 to 142, and 143 to 170 days of age) shown as the phase before second vaccination with Improvac® (before V2), the phase after immunization with Improvac® (after V2), and separated first (day 115 to 142) and second (day 143 to 170) 4-week phases following V2. Pigs were fed diets containing low (LNE), medium (MNE), or high (HNE) NE content. BW, body weight; V2, second vaccination with Improvac® NE, net energy. ^a−b, A−C^Least square means with different superscripts differ (p < 0.05). ^1^RSD, residual standard deviation, which is the root-mean-square error that applies to the whole model. ^2^In periods before and after V2, the effect of dietary treatment (Diet) and litter origin were included in the model, whereas for the 2 separate 4-week phases in the period after V2, the effect of phase and its interaction with dietary treatment (Diet × Phase) were considered. Effect of phase was always significant (p < 0.01) and is therefore not reported in the table. ^3^Adjusted for 89% dry matter content of the diet. ^4^Calculated using the dietary net energy content calculated from Exp. 2. ^5^For average daily gain, dressing percentage was added to the model, in order to eliminate the influence of energy accumulated in the gut content and gut tissue (23)*.

The effectiveness of immunocastration in the growth trial (Exp. 2) was assessed considering backfat concentrations of androstenone and skatole and testicular weight at slaughter. With the use of these parameters, no non-responders to immunocastration were identified. Androstenone and skatole concentrations averaged 0.27 ± 0.08 and 0.05 ± 0.03 μg/g of liquid fat, which is just above the detection limit of the determination method. Weight of testes with epididymis averaged 247 ± 147 g (*p* = 0.78; results not shown), and the gonadosomatic index averaged 0.204 ± 0.132 (*p* = 0.78; results not shown).

### Nutrient Digestibility of Experimental Diets (Experiment 1)

The results of the digestibility trial (Exp. 1) presented in Table 3 reveal a lower (*p* < 0.05) digestibility of OM, CP, ether extract, and energy in the LNE diet, in accordance with its greater fiber content. On the other hand, greater crude fiber digestibility was noted for the LNE diet (*p* < 0.01), whereas NDF and ADF digestibility were not affected (*p* = 0.83 and 0.12) by diet composition. Similarly, nitrogen intake and retention were similar for the LNE and HNE dietary treatments (*p* = 0.18 and 0.34), whereas greater nitrogen fecal losses (*p* < 0.01) and lower nitrogen urinary losses (*p* = 0.04) were observed for the LNE treatment group. Consistent with the objective of the study, the experimental diets differed in their energy contents; DE (*p* = 0.01), ME (without the contribution of CH_4_; *p* = 0.02), and calculated NE (*p* < 0.01) contents of HNE and LNE diets were 13.9 vs. 12.7, 13.4 vs. 12.3, and 10.4 vs. 9.3 MJ/kg, respectively (Table 3).

**Table 3:** Digestive utilization of nutrients, nitrogen balance, and energy values of diets (Exp. 1^a^).

	**Diet** ^*****b*****^			
**Item**	**LNE**	**HNE**	**RSD^***c***^**	***p*-Value^***d***^**
BW at first stage (kg)	47.1	44.7	6.4	0.71
ADFI during first stage^*e*^ (kg of feed)	2.28	1.94	0.29	0.29
BW at second stage (kg)	67.7	70.5	7.2	0.70
ADFI during second stage^*e*^ (kg of feed)	2.73	2.62	0.34	0.75
**Digestibility coefficients (%)**				
Organic matter	81.6	87.2	1.0	0.02
Crude protein	73.2	82.2	2.1	0.02
Ether extract	58.3	69.1	2.5	0.02
Crude fiber	44.7	29.7	3.7	0.02
Neutral detergent fiber	52.1	52.8	4.2	0.83
Acid detergent fiber	45.0	38.9	3.6	0.12
Energy	78.9	85.1	1.2	0.02
**Energy values**^*e*^ **(MJ/kg of feed)**				
DE	12.70	13.87	0.20	0.01
ME^*f*^	12.31	13.42	0.25	0.02
NE^*g*^	9.29	10.38	0.15	<0.01
**N balance (g/day)**				
Intake	60.6	58.4	1.7	0.18
**Excretion**				
In feces	16.2	10.3	0.8	<0.01
In urine	13.6	20.5	2.2	0.04
Retained	30.7	27.6	3.9	0.34
N retention (% of digested N)	69.4	59.2	6.5	0.14

*BW, body weight; ADFI, average daily feed intake; DE, digestible energy; ME, metabolizable energy; NE, net energy; N, nitrogen*.

a *Exp. 1 consisted of a digestibility trial and nitrogen balance study, and it was performed using 4 pigs (immunocastrated with Improvac® at age of 77 and 105 days) fed the 2 extreme experimental diets of Exp. 1 during 2 successive periods (first prior and second after immunization with Improvac®). Each period consisted of one adaptation sub-period (14 days) and a subsequent collection sub-period (7 days) so that 2 pigs were fed each diet at each stage*.

b *Diets with low (LNE) or high (HNE) NE content*.

c *RSD, residual standard deviation*.

d *Values were tested for the effect of diet; results are least-squares means; the measurement was considered repeated per period*.

e *Adjusted for 89% DM content*.

f *It should be noted that ME did not account for the loss of energy as CH_4_*.

g *Calculated according to Noblet et al. (8); average of equations 3, 4, and 5*.

### Feed Intake and Performance (Experiment 2)

No effect of the diet (Table 2) on BW at any stage of the experiment was detected. The period after effective immunization significantly affected NE feed intake and growth performance (*p* < 0.01; Table 2), whereas the phase and dietary treatment interaction after immunization was significant only for backfat gain (*p* < 0.01; Table 2). Diet composition did not affect the ADFI in any period of the experiment (*p* > 0.05; data not shown), and NE intake was affected by the changes in diet NE concentration, but this effect was significant only in P2. To be more precise, greater daily NE intake expressed as megajoules per kilogram of BW^0.60^ was noted between the HNE dietary group compared with the MNE dietary group in P2 (*p* = 0.03; Table 2). There was no effect (Table 2) of dietary NE concentration on ADG and G:NE in any period of the experiment. A difference in backfat gain was observed in the second phase after immunization (day 143 to 170), where greater (*p* < 0.01; Table 2) backfat gain was noted for the HNE group compared with the MNE and LNE groups, which was also reflected as a tendency (*p* = 0.13; Table 2) toward greater backfat gain in P2 (day 115 to 170) in the HNE group.

**Figure 2:**
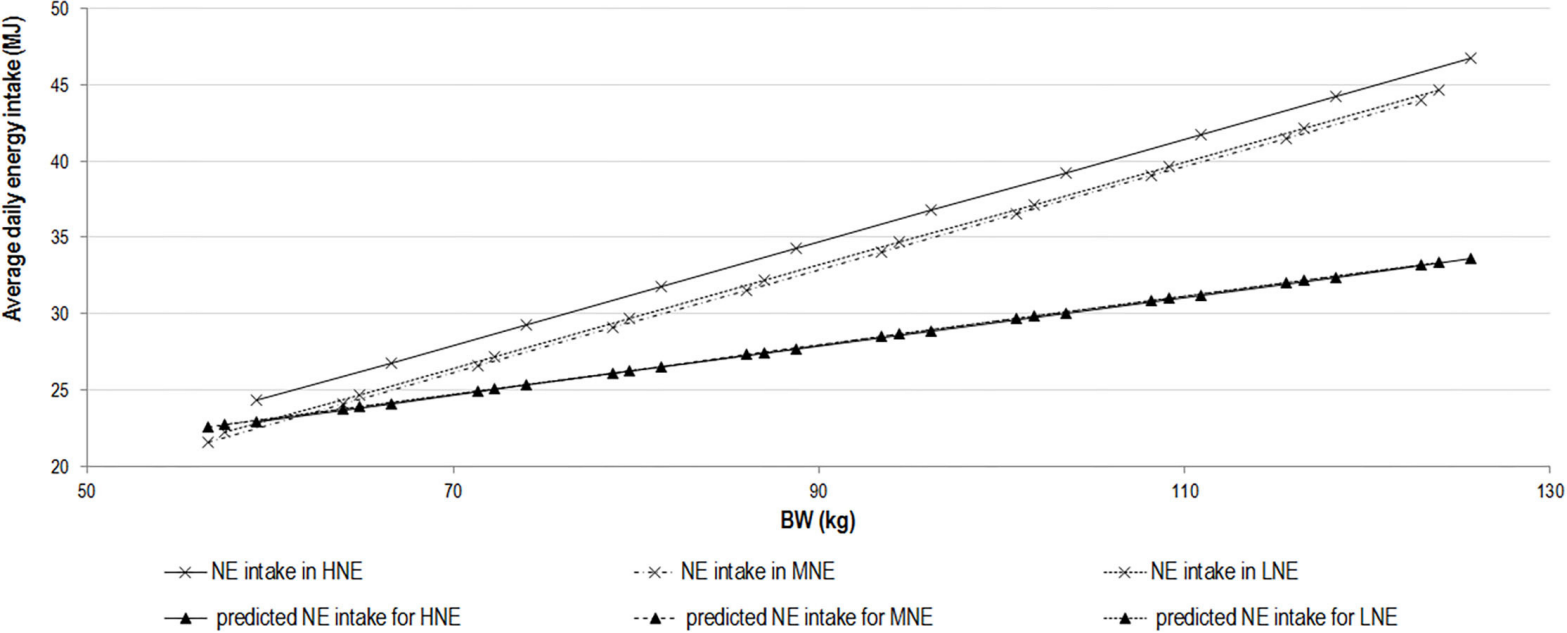
Relationship between daily net energy intake and body weight of growing–finishing immunocastrated pigs. Relationship between daily net energy (NE) intake and body weight of growing–finishing immunocastrated pigs (vaccinated with Improvac®, Zoetis at the age of 77 and 112 days) on experimental diets containing 10.4 (LNE) and 11.0 (MNE) MJ/kg of DM in comparison with the standard diet containing 11.6 (HNE) MJ/kg of fed diets. Predicted NE intake was calculated as 4.09 × BW^0.43^. LNE, low NE; MNE, medium NE; DM, dry matter; BW, body weight.

To further elucidate the effect of energy intake and period after V2, a detailed analysis of the energy intake data was performed, based on the cumulative consumption of the experimental diets shown in Figure 2. The slopes of the relationships between cumulative energy intake and BW were tested for the effects of phase (prior = P1 or after immunization = P2) and diet using the 1.43 exponent applied to BW. Regardless of diet, the change in cumulative energy intake per kg BW gain never differed between periods for cumulative ME and NE intake (Table 4). In each period (before and after V2), the change in cumulative ME intake per kg BW gain tended to differ (*p* = 0.05 and 0.06) between the two extreme diets, i.e., LNE and HNE, whereas the change in cumulative NE intake per kg BW increase did not differ between periods and diets and averaged 2.86 MJ/kg BW^1.43^-463 MJ (Table 4). From the intercept and the slope of the relationship calculated for the first period, it can be estimated that BW at zero cumulative consumption (i.e., at the beginning of the experiment) averaged 34.9 kg (SD = 1.2 kg); the latter is close to the values reported in Table 2 at 84 days of age.

**Table 4:** Relationship between cumulative dietary energy intake and body weight in immunocastrated pigs.

	**Cumulative ME intake, MJ**			**Cumulative NE intake, MJ**		
	**Before V2**	**After V2**	***p*-Value^**1**^**	**Before V2**	**After V2**	***p*-Value^**1**^**
Exponent applied to BW (c)	1.43	0.47	1.43	0.47
**Slope, MJ/kg BW** ^**c**^						
HNE	3.47	3.72	0.44	2.69	2.88	0.43
MNE	3.61	3.75	0.61	2.76	2.87	0.61
LNE	4.20	3.81	0.28	3.17	2.89	0.29
***p*** **-Values for test difference between** ^**2**^						
HNE and LNE	0.06	0.05		0.11	0.68	
HNE and MNE	0.63	0.53		0.65	0.66	
MNE and LNE	0.17	0.26		0.22	0.65	
**Calculated intercept, MJ** ^**3**^						
HNE	−537	−605		−416	−469	
MNE	−562	−600		−430	−459	
LNE	−721	−617		−544	−466	
Residual standard deviation, MJ^4^	75			57		

*Relationship between cumulative dietary energy intake (in MJ) before and after effective immunocastration (V2) and BW in growing–finishing immunocastrated pigs (n = 41) according to diet energy content. Vaccination with Improvac® (2 ml, s.c. application, Zoetis) was performed at the age of 77 (V1) and 112 (V2) days. Parameters are estimated for the generalized relationship between cumulative GE/DE/ME/NE intake (MJ) and body weight (kg) before and after second vaccination for immunocastration and different diets (HNE, high NE diet; MNE, medium NE diet; and LNE, low NE diet) in the form*:

*If age <112*:

*
$y=a_{diet\;before\;V2}\times BW^{c_{diet}}+b_{pig}$
*

*If age ≥ 112*:

*
$y=a_{diet\;after\;V2}\times BW^{c_{diet}}+\left(a_{diet\;before\;V2}-a_{diet\;after\;V2}\right)\times BW_{age\;=\;112}+b_{pig}$
*

*a_diet before V2_ and a_diet after V2_ are the slopes of the relationship for each diet, before and after V2, respectively; c_diet_ is the exponent applied to BW for each diet, BW_age_ = 112 is the BW at 112 days of age; and b_pig_ is the intercept of the relationship for each pig. BW, body weight; ME, metabolizable energy; NE, net energy; GE, gross energy; DE, digestible energy. ^1^p-Values of the residual sum of squares of the reduced model (single exponent c for all diets, or single exponent c for all diets plus single slope for both periods—prior and after V2 for each diet) are equal to those of the full model. Hypothesis for which p > 0.05 is indicative that the reduced model is adequate to describe the data. ^2^p-Values of the residual sum of squares of the reduced model (single exponent c for all diets plus single slope for 2 diets at each period—prior and after V2) are equal to those of the full model. Hypothesis for which p > 0.05 is indicative that the reduced model is adequate to describe the data. ^3^Intercept calculated as the average per diet of individual b_pig_ values for P1 (prior V2) and as the average per diet of individual [(a_diet before V2_ – a_diet after V2_) × BW_age_ = 112 + b_pig_] for P2 (after V2). ^4^Residual standard deviation for the full model calculated as the square root of residual sum of squares divided by residual degrees of freedom*.

*^c^ Exponent applied to BW*.

### Morphology, Mitotic Cell Count, and Skatole and Indole Production in Intestinal Segments (Experiment 2)

The effect of a reduction in NE concentration of the diet on the intestinal histo-morphological features (*p* < 0.01; Table 5) was evident in the jejunum, ileum, ascending, and descending colon, whereas the mucosa of the duodenum and cecum was not affected (Table 5). To be more specific, greater villus surface area, villus width, villus height, villus perimeter, crypt depth, and thickness of the intestinal mucosa were noted for pigs fed LNE diet (*p* < 0.01; Table 5) than those fed HNE diet, with those fed MNE diet being intermediate. In the ileum, crypt depth increased with dietary NE content reduction (*p* < 0.01; Table 5), whereas the thickness of the intestinal mucosa was increased in the LNE and MNE dietary groups compared with the HNE group (*p* < 0.01; Table 5). Despite the change in histo-morphological characteristics of the intestinal mucosa, dietary treatment did not appear to be associated with the enterocyte proliferation (i.e., PCNA-positive cell number) in any of the intestinal sections studied (*p* > 0.05; Table 5).

**Table 5:** Effect of changes in dietary net energy concentration on intestinal morphology and mitotic cell count.

	**Treatment**				
**Item**	**LNE**	**MNE**	**HNE**	**RSD^**1**^**	***p*-Value**
*n* of pigs	6	6	6		
**Duodenum**					
Villus surface area, × 10^4^ μm^2^	29.0	28.3	22.5	6.0	0.16
Villus width, μm	214	211	174	30.6	0.07
Villus height, μm	433	423	411	58.0	0.81
Villus perimeter, μm	1,099	1,036	1,050	123.3	0.66
Crypt depth, μm	563	511	503	44.7	0.07
Thickness of intestinal mucosa, μm	997	934	914	66.7	0.11
Villus height/crypt depth	0.78	0.83	0.83	0.4	0.75
PCNA, number of positive cells	7.5	8.0	7.3	1.5	0.75
**Jejunum**					
Villus surface area, × 10^4^ μm^2^	48.0^c^	32.8^b^	23.9^a^	4.0	<0.01
Villus width, μm	262^b^	222^ab^	187^a^	25.2	<0.01
Villus height, μm	588^b^	471^a^	404^a^	44.4	<0.01
Villus perimeter, μm	1,461^c^	1,194^b^	1,010^a^	83.6	<0.01
Crypt depth, μm	414^c^	351^b^	287^a^	22.9	<0.01
Thickness of intestinal mucosa, μm	1,002^c^	822^b^	692^a^	50.4	<0.01
Villus height/crypt depth	1.42	1.36	1.42	0.2	0.86
PCNA, number of positive cells	7.6	7.4	7.3	1.5	0.92
**Ileum**					
Villus surface area, × 10^4^ μm^2^	23.9	23.3	16.4	7.6	0.26
Villus width, μm	189	191	161	33.7	0.31
Villus height, μm	387	387	313	78.0	0.26
Villus perimeter, μm	1,068	991	812	157.3	0.08
Crypt depth, μm	413^c^	385^b^	284^a^	18.1	<0.01
Thickness of intestinal mucosa, μm	801^b^	772^b^	597^a^	78.5	<0.01
Villus height/crypt depth	0.94	1.01	1.10	0.2	0.58
PCNA, number of positive cells	6.8	7.5	7.4	1.6	0.74
**Cecum**					
Crypt depth, μm	404	394	412	52.0	0.83
PCNA, number of positive cells	6.9	6.7	6.5	1.3	0.77
**Colon ascendens**					
Crypt depth, μm	504^b^	406^a^	389^a^	23.5	<0.01
PCNA, number of positive cells	12.0	13.7	13.3	3.1	0.67
**Colon descendens**					
Crypt depth, μm	535^b^	479^ab^	433^a^	38.4	<0.01
PCNA, number of positive cells	9.0	9.0	9.7	2.1	0.85

*Effect of changes in dietary net energy concentration on intestinal morphology and mitotic cell count was evaluated in growing–finishing immunocastrated pigs, fed diets containing low (LNE), medium (MNE), or high (HNE) NE content. Vaccination with Improvac® (2 ml, s.c. application, Zoetis) was performed at age of 77 and 112 days. NE, net energy; PCNA, proliferating cell nuclear antigen. ^a−c^Least squares means within a row with different superscripts differ (p < 0.05). ^1^RSD, residual standard deviation, which is the root-mean-square error that applies to the whole model*.

Indole concentration in the ascending colon was decreased (*p* < 0.01; Table 6) by decreasing dietary NE concentration, while skatole concentration in the cecum was increased with NE reduction (*p* < 0.01; Table 6) and tended to decrease with NE reduction in the descending colon (*p* = 0.07; Table 6).

**Table 6:** Effect of changes in dietary net energy concentration on intestinal skatole and indole production.

	**Treatment**				
**Item**	**LNE**	**MNE**	**HNE**	**RSD^**1**^**	***p*-Value**
*n* of pigs	14	13	14		
**Indole in intestinal content**, **μg/g**					
Cecum	8.13	14.0	17.7	7.2	0.10
Colon ascendens	3.9^a^	10.8^b^	19.7^b^	5.5	<0.01
Colon descendens	14.0	6.6	9.9	6.8	0.20
**Skatole in intestinal content**, **μg/g**					
Cecum	21.9^b^	5.5^a^	4.3^a^	5.8	<0.01
Colon ascendens	15.4	16.5	22.9	21.5	0.35
Colon descendens	5.5	9.7	17.9	8.2	0.07

*Effect of changes in dietary net energy concentration on skatole and indole production was evaluated in segments of the large intestine of growing–finishing immunocastrated pigs, fed diets containing low (LNE), medium (MNE), or high (HNE) net energy content. Vaccination with Improvac® (2 ml, s.c. application, Zoetis) was performed at age of 77 and 112 days. ^a−b^Least squares means within a row with different superscripts differ (p < 0.05). ^1^RSD, residual standard deviation, which is the root-mean-square error that applies to the whole model*.

### Carcass Characteristics and Fat Deposition (Experiment 2)

In agreement with the lack of effect of diet on BW, no effect (*p* = 0.20) was observed on HCW or weight of major carcass parts (data not shown). However, it should be noted that the numerical variation in dressing percentage between dietary treatment groups, although not significant (*p* = 0.11), was quite high (1.5% and 0.9% points lower for the LNE and MNE groups, respectively, compared with the HNE group; Table 7), consistent with the effect of dietary treatment on backfat gain measured *in vivo* by ultrasound (*p* = 0.02; Table 7) and as the area of fat covering the longissimus muscle (*p* = 0.05; Table 7) was lower in LNE and MNE pigs than in pigs fed HNE diet. Decreased subcutaneous fatness was reflected in greater lean meat content (*p* = 0.04; Table 7) of LNE compared with HNE pigs.

**Table 7:** Effect of changes in dietary net energy concentration on carcass and meat quality traits.

	**Treatment**				
**Item**	**LNE**	**MNE**	**HNE**	**RSD^**1**^**	***p*-Value**
*n* of pigs	14	13	14		
**Measurements before and at slaughter**					
Live weight, kg	125.0	123.2	129.9	11.1	0.29
Final backfat thickness, mm	15.7^a^	16.1^a^	18.5^b^	2.5	0.02
Hot carcass weight^2^, kg	96.9	96.3	102.6	9.6	0.20
Dressing percentage, %	77.5	78.1	79.0	1.8	0.11
**Measurements on carcass**					
Lean content^3^, %	56.8^b^	56.3^ab^	54.8^a^	1.8	0.04
Leaf fat, %	1.4	1.4	1.5	0.3	0.57
Backfat thickness^4^, mm	18.2	18.1	18.9	4.4	0.88
NIMF^5^, %	22.7	21.2	24.7	4.1	0.30
LM area^6^, cm^2^	51.1	51.3	52.5	5.4	0.77
Fat above LM area^6^, cm^2^	17.4^a^	18.2^ab^	20.5^b^	3.2	0.05
**Meat quality**					
LM intramuscular fat, %	1.54	1.45	1.68	0.62	0.66

*Effect of changes in dietary net energy concentration was evaluated on selected carcass and meat quality traits in growing–finishing immunocastrated pigs. Pigs were fed diets containing low (LNE), medium (MNE), or high (HNE) NE content. Vaccination with Improvac® (2 ml, s.c. application, Zoetis) was performed at age of 77 and 112 days; slaughter was performed in two batches within 5 days, at an average age of 172 days. LM, longissimus muscle; NE, net energy. ^a−b^Least squares means within a row with different superscripts differ (p < 0.05). ^1^RSD, residual standard deviation, which is the root-mean-square error that applies to the whole model. ^2^With head. ^3^Calculated from backfat (F34) and muscle (M34) thicknesses measured on hot carcass: 62.19 – 0.729 × F34 + 0.144 × M34 (http://uniporc-ouest.com). ^4^Backfat thicknesses measured at the level of the last rib. ^5^NIMF, neck intermuscular fatness, assessed by image analysis (5). ^6^LM area and fat over LM area were assessed by image analysis (5)*.

## Discussion

In IM pigs, rapid changes in hormonal status 10 to 15 days after immunization (2) result in increased voluntary feed intake, greater ADG (3, 24), and altered energy metabolism associated with increased lipid deposition (25, 26). Consequently, this leads to greater BFT and lower lean carcass content [as determined by the meta-analytical studies (3, 4)], in parallel with the effective elimination of boar taint compounds in adipose tissue. However, most of the studies conducted to date dealt with pigs slaughtered 4 to 6 weeks after immunization, whereas little is known about what happens when the delay between immunization and slaughter is further extended. In the present study, this delay was increased up to 8 weeks without any adverse effects on meat boar taint: taking into account androstenone and skatole levels in the backfat together with the testicular weight, all pigs could be considered fully immunized. Similarly, Kubale et al. (27) observed no evidence of functional or morphological recovery of testicular function within 8 weeks after immunization.

The high energy digestibility observed in Exp. 1 with a low fiber (i.e., high starch) diet is to be expected and agrees with previous studies (28–30). It also confirms that fiber-rich feedstuffs can be successfully used as an energy diluting factor in growing–finishing diets because of components with a low digestive utilization (31). The inclusion of dietary fiber also increases the endogenous losses, resulting in a perceived decrease in the digestion of energy and nutrients in monogastric animals (32). Indeed, by increasing NDF content (plus 41 and 81 g/kg DM in MNE and LNE diets, respectively), the calculated NE content of the diets in the present study was reduced by approximately 5 and 10% in MNE and LNE diets. On the other hand, in Exp. 1, dietary fiber intake resulted in increased digestibility of fiber components. This may be explained by the ability of the intestinal mucosa to adjust to a high-fiber diet with a gradual change in microbial fermentation as also confirmed in Exp. 2 by the results of skatole and indole production Additionally, dietary fiber fermentation by the intestinal microbiota results in short-chain fatty acid (SCFA) production (33), which can directly contribute to the nutritional value of meal as an energy source (34), but with a lower energetic efficiency than starch and lipid (35). As indicated by Jin et al. (36), the inclusion of 10% high-fiber source in diets of growing pigs for 14 days caused an increased width of villi and depth of the crypts in the jejunum and ileum. The same study also showed that high dietary fiber intake increased the rate of cell proliferation and crypt depth in the colon. However, this was not evident in the present study at the level of the small intestine, despite the changes in histo-morphological features, which may be since intense changes in intestinal morphology may have occurred earlier (immediately after the change in diet, e.g., at the age of 84 days).

In the present study, skatole level increased with the NE reduction in the cecum but tended to decrease with NE reduction in the descending colon. The reason for the differential response in different intestine segments may be related to the progressive decrease in the flow of digesta toward the distal colon that changes the fermentation metabolite and bacterial profile (37, 38). Nevertheless, an overall reduction of skatole with decreased NE and increased amount of dietary fiber can be observed. Similarly, Li et al. (39) demonstrated that the addition of sugar beet pulp, one of the ingredients used in the present study to reduce the energy level of the experimental diet, had a significant inhibitory effect on skatole formation in the intestine. The reasons may be several, from increased intestinal passage rate (37) and increased fecal water content diluting and lowering skatole accumulation rate to serving as a fermentable energy source, preventing amino acid (i.e., tryptophan) fermentation and skatole production (40). In the absence of fermentable carbohydrates as an energy source, microbial fermentation shifts to amino acids and uses the carbon skeleton of amino acids as an energy source (41), which also leads to increased production of intestinal skatole (42) as an amino acid (tryptophan) metabolite. On the other hand, in the presence of energy from fermentable carbohydrates, the resident microbes in the colon retain more amino acids for their growth (43). Along with decreasing skatole, indole production behaved oppositely (decreased with NE reduction in the ascending colon). Although skatole and indole production could be positively correlated due to their common precursor (42), the synthesis of both compounds is pH-dependent [indole-forming bacteria favor basic and skatole-producing bacteria acidic conditions (44)] and can be influenced in different ways by dietary components (45).

Pigs are generally able to adapt voluntary feed intake to dietary characteristics, and energy density is usually the first determinant of ADFI as long as the maximum capacity of the digestive tract is not reached (23, 46). However, the results of the present study are evidence of more complex interactions due to the inclusion of dietary fiber in the diet, which can be continually fermented by intestinal microbiota, providing a stable energy source and thus prolonged satiety (47), and changes in lipid metabolism (enhanced lipid deposition and lipogenic activity) after immunocastration (25, 48). In agreement with the results of Zeng et al. (49), reduction of diet NE concentration did not significantly affect the ADFI of IM pigs after immunization, which might indicate that the pigs have already reached their maximal feed intake capacity or that the dietary source used in the lowest NE diets had a marked effect on pig satiety (23, 50). The latter might be due to increased intestinal volume as a consequence of greater bulk density and water-holding capacity of a high-fiber diet (51) and, additionally, due to the effect of SCFA on appetite regulation and long-term satiety (52). Since the ADFI of IM pigs in the first weeks after immunization is greater than in their surgically castrated counterparts [by ~100 g/day; (1, 3, 24)], the physical limits for ADFI may be reached independently of the diet energy content. In the present study, a tendency toward lower NE intake was noted for LNE and MNE diet in the second 4-week period after immunization. However, only pigs consuming the MNE diet, expressed on a metabolic BW basis (MJ/kg BW^0.60^ per day), reached significantly lower NE intake after immunization. This might indicate a nonlinear effect of reducing diet energy density on the regulation of energy intake or that LNE pigs still compensated for the lower NE dietary content with greater ADFI (which was numerically, but not statistically greater), whereas this was not the case for MNE pigs. It was not possible to measure feeding behavior in our trial, but our results on DFI and the influence of dietary fiber on fermentation and satiety suggest that modifications on feeding behavior should be considered to explain the difference between treatments. Additionally, the variability of NE intake (MJ/kg BW^0.60^ per day) is much smaller, implying that the variability of daily NE intake and ADFI is partly explained by the variability in BW, which was numerically lower for the LNE and MNE groups. However, when cumulative energy intake is considered as a function of BW, small differences in the slopes for cumulative DE and ME intake can be observed (Table 4) whereas no difference was observed in the slope of the relationship between cumulative NE intake and BW between periods and diets. It can thus be considered that maximal daily NE intake (MJ), which does not depend on diet characteristics, was achieved regardless of the dietary energy content and can be described as the first derivative according to BW of the average prediction equation for cumulative NE intake. It corresponds to 4.09 MJ/kg × BW^0.43^ MJ when BW varied from 40 to 125 kg.

To eliminate the influence of differential gut content and visceral tissue related to the ADFI differences (23), the ADG was adjusted for dressing percentage, and it remained unaffected by 5% or 10% reduction of the diet NE concentration in the present study. When even stricter quantitative feed restriction was applied in IM pigs, a decrease in ADG was reported (by 20 and 12% at 78 and 85% of *ad libitum* feed intake, which corresponds to −6.9 and −4.6 MJ consumed NE per day, respectively), with no improvement in G:F (7). Due to energy dilution in the present study, growth efficiency was evaluated on NE basis as G:NE, to express energy requirements and dietary energy values on the same scale (53). In accordance with the results of Quiniou and Noblet (7) in barrows, G:NE was similar in all 3 dietary groups, indicating that changes in diet NE concentration did not influence the growth rate of IM pigs, although the partitioning of energy was different among dietary groups. Namely, in accordance with the objective of the study, changes in diet NE concentration (10% compared with the standard 10.4 MJ NE/kg feed) resulted in a reduced backfat deposition. The difference between the 3 dietary treatments in the rate of backfat gain was significant during the second 4-week period following immunization, resulting in a 28% lower rate of backfat deposition. In general, lipid deposition in pigs increases gradually with BW (54), but IM pigs are characterized by an intense increase in lipid deposition after immunization and a subsequent increase in the ratio between lipid and protein deposition [LD:PD; (26)]. It was therefore expected, and demonstrated in the present study, that the difference in backfat gain due to the reduced dietary NE content would become noticeable only after a long period after immunization, providing an additional argument that the pigs were not able to fully adjust their feed intake to achieve a constant energy intake across treatments.

The results of carcass traits confirm the results observed for performance. In agreement with a similar study in barrows (7), the carcass weight of IM pigs in the present study was not affected by the reduction in diet NE concentration, whereas lean content was improved. Weights of prime cuts were similar in all 3 dietary treatment groups, whereas the response of fat depots varied. The subcutaneous fat depot was significantly reduced with reduction of the NE content of the feed, whereas smaller (numerical only) reduction was observed for leaf fat and intramuscular fat. Intramuscular fat content was below the level of 2% to 3% required to impact flavor and juiciness to pork meat (55) in all dietary groups, most likely due to the genetic lines used. Additionally, the lack of effect of NE reduction on leaf fat deposition is expected given the relative growth rate of different fat depots, which serve as a source of energy. As demonstrated in barrows, leaf fat, located within the abdominal cavity, exhibits the most rapid response to prolonged dietary stimuli, followed by subcutaneous and intermuscular (and intramuscular) fat deposition (56–58). In the present study, pigs were fed on an *ad libitum* basis, which means that they were not in energy deficiency, because pigs can adapt feed intake to the energy density of the feed (23, 46). We can then assume that eventual additional energy to the one required for basal metabolic needs consumed by pigs on the LNE diet was firstly deposited to leaf fat and less was available for other tissues. Regarding the effect of quantitative feed restriction in IM pigs, literature reports either no significant effect on carcass leanness and fat deposition (7, 59) or only an effect on leaf fat weight (5). The lack of effect of restricted feeding on carcass leanness in the abovementioned studies could be associated with a shorter duration of the restriction. As shown in the present study, backfat deposition significantly increases only after a substantial time post-immunization (4 to 8 weeks after V2).

Taken together, the results of the present experiment confirm that IM pigs are energetically very efficient and suggest that energy dilution in the growing and finishing diet offered *ad libitum* to IM pigs limits the subcutaneous fat deposition. From a practical perspective, this indicates that in the countries where BFT is a determinant of carcass value (i.e., European Union and the United States), it might be reasonable to use feed restriction (quantitative or qualitative), especially if an extended time between immunization and slaughter is anticipated as well as when pigs are fattened to heavy weights for high-quality dry-cured products, where excessive adiposity must be avoided due to market demand.

The advantage of immunocastration resides in the long-term boar-like performance of pigs; however, after the immunization, feed intake is considerably increased, which diminishes the advantages in performance. Overall, our results demonstrate that by reducing the NE concentration of the diet by up to 10% with high-fiber content ingredients, similar energy efficiency (gain to NE intake ratio) may be achieved due to effective adaptation to the high-fiber diet, as indicated by histo-morphological characteristics of the intestinal mucosa. Moreover, reducing dietary NE concentration lowers intestinal skatole production. This technique provides an advantage in terms of reduced lipid deposition and improved leanness, without affecting growth rate in IM pigs after immunization, which is particularly important when the BFT is a determinant of carcass value and IM pigs are fattened to higher weights (e.g., in heavy pig production) or when a longer delay between immunization and slaughter is practiced.

## Data Availability Statement

The raw data supporting the conclusions of this article will be made available by the authors, without undue reservation.

## Ethics Statement

The animal study was reviewed and approved by the Ethics Committee of the French Ministry of Agriculture, certificate of authorization for an experiment on live animals No. 35-110 was issued to Etienne Labussière.

## Author Contributions

NB-L and EL contributed to the conception, design of the study, and performed the statistical analysis. NB-L, MČ-P, MŠ, VK, and EL performed the sample collection and analysis. NB-L wrote the first draft of the manuscript. VK and MČ-P wrote sections of the manuscript. All authors contributed to manuscript revision and read and approved the submitted version.

## Funding

The authors acknowledge the financial support of the Slovenian Research Agency (Ph.D. fellowship of NB-L, projects Z7-9416 and L4-5521, programs P4-0133 and P4-0053) and Rennes Metropole and Slovene Human Resource Development and scholarship funds for Ph.D. student mobility.

## Conflict of Interest

The authors declare that the research was conducted in the absence of any commercial or financial relationships that could be construed as a potential conflict of interest. The reviewer DK declared a past co-authorship with several of the authors, MČ-P, MS, and NB-L, to the handling editor.

## Publisher's Note

All claims expressed in this article are solely those of the authors and do not necessarily represent those of their affiliated organizations, or those of the publisher, the editors and the reviewers. Any product that may be evaluated in this article, or claim that may be made by its manufacturer, is not guaranteed or endorsed by the publisher.

## Abbreviations

ADF: acid detergent fiber
ADFI: average daily feed intake
ADG: average daily gain
BFT: backfat thickness
BW: body weight
CP: crude protein
DE: digestible energy
DFI: daily feed intake
DM: dry matter
EM: entire males
Exp.1: experiment 1
Exp. 2: experiment 2
G:F: gain to feed ratio
G:NE: gain to net energy ratio
GE: gross energy
HCW: hot carcass weight
HNE: high net energy
IM: immunocastrated pigs
LNE: low net energy
ME: metabolizable energy
MNE: medium net energy
NDF: neutral detergent fiber
NE: net energy
OM: organic matter
SCFA: short-chain fatty acid
V1: first vaccination
V2: second vaccination.
